# Hydrogen Sulfide as a Potential Therapeutic Target in Fibrosis

**DOI:** 10.1155/2015/593407

**Published:** 2015-05-11

**Authors:** Shufang Zhang, Chuli Pan, Feifei Zhou, Zhi Yuan, Huiying Wang, Wei Cui, Gensheng Zhang

**Affiliations:** ^1^Department of Cardiovascular Medicine, Second Affiliated Hospital, Zhejiang University School of Medicine, Binjiang Branch, Hangzhou 310009, China; ^2^Department of Critical Care Medicine, Second Affiliated Hospital, Zhejiang University School of Medicine, Hangzhou 310009, China; ^3^Department of Critical Care Medicine, Ningbo Medical Center, Lihuili Hospital, Ningbo University, Ningbo 315041, China; ^4^Department of Respiratory Medicine, Fenghua People's Hospital, Fenghua, Ningbo 315000, China; ^5^Department of Allergy, Second Affiliated Hospital, Zhejiang University School of Medicine, Hangzhou 310009, China

## Abstract

Hydrogen sulfide (H_2_S), produced endogenously by the activation of two major H_2_S-generating enzymes (cystathionine *β*-synthase and cystathionine *γ*-lyase), plays important regulatory roles in different physiologic and pathologic conditions. The abnormal metabolism of H_2_S is associated with fibrosis pathogenesis, causing damage in structure and function of different organs. A number of *in vivo* and *in vitro* studies have shown that both endogenous H_2_S level and the expressions of H_2_S-generating enzymes in plasma and tissues are significantly downregulated during fibrosis. Supplement with exogenous H_2_S mitigates the severity of fibrosis in various experimental animal models. The protective role of H_2_S in the development of fibrosis is primarily attributed to its antioxidation, antiapoptosis, anti-inflammation, proangiogenesis, and inhibition of fibroblasts activities. Future studies might focus on the potential to intervene fibrosis by targeting the pathway of endogenous H_2_S-producing enzymes and H_2_S itself.

## 1. Introduction

Hydrogen sulfide (H_2_S), known for decades as a noxious and toxic gas, has been recognized recently as a third gasotransmitter, together with its two counterparts nitric oxide (NO) and carbon monoxide (CO) [1]. H_2_S plays important regulatory roles in different physiologic and pathologic conditions, including hypertension, angiogenesis, neurodegenerative diseases, inflammation, and metabolic syndrome, to name a few [2]. H_2_S is endogenously generated in various tissues through the transsulfuration pathway by two pyridoxal-5′-phosphate-dependent enzymes, cystathionine *β*-synthase (CBS) and cystathionine *γ*-lyase (CSE), with L-cysteine and homocysteine (Hcy) as the substrates. Besides, 3-mercaptopyruvate sulfurtransferase (MST) may produce H_2_S through the cooperation with cysteine aminotransferase [1, 2].

Fibrosis is a complicated process, in which the tissue repair after injury is too strong or out of control, and thus results in excessive formation of fibrous connective tissue. This process could be triggered by various factors such as inflammation, immunity, toxicant, ischemia, or hemodynamic changes [3]. Fibrosis can occur in multiple tissues or organs including lung, heart, liver, and kidney. The main pathological changes of fibrosis are the increased fibrous connective tissue and decreased parenchymal cells, which can cause organ structural damage, functional decline, and even failure, seriously threatening to human health [4].

Previously, H_2_S was considered as a toxic environmental pollutant, which can cause early lung damage, chronic lung inflammation, and pulmonary fibrosis [5, 6]. However, the physical role of H_2_S in the development of fibrosis has attracted significant attention recently. Numerous studies have demonstrated that endogenous and exogenous H_2_S play a critical role in the development of fibrosis in lung [7–10], liver [11–13], kidney [14, 15], and heart [16–18]. This review focuses on the protective roles of H_2_S in fibrosis pathogenesis.

## 2. Altered Endogenous H_**2**_S/Its Producing Enzymes in Fibrosis Pathogenesis 

The H_2_S-producing enzymes/endogenous H_2_S pathways are involved in the development of fibrosis. The deficiency in endogenous CBS/H_2_S or CSE/H_2_S system is responsible for fibrosis [8, 19]. Downregulation of CBS and CSE expression/activity and decreased plasma H_2_S levels were observed in patients with hepatocirrhosis [20–23]. And animal models of various organ fibrosis demonstrated the significant decrease of the endogenous H_2_S level in plasma and tissues and the H_2_S-producing enzymes, whereas the administration exogenous H_2_S could inhibit the fibrosis development [9, 13, 15, 18, 19, 24, 25].

### 2.1. H_2_S and Pulmonary Fibrosis

Pulmonary fibrosis, a chronic and progressive interstitial lung disease, is triggered by various factors like organic and inorganic particles, chemicals, radiation, and infections. Its common steps are fibroblastic foci formation, exaggerated extracellular matrix (ECM) deposition, and eventually leading to the destruction of the lung parenchymal architecture [26–28]. The H_2_S-producing enzymes (CSE, CBS, and/or MST) are expressed in human and animal lungs [29], and the physiologic plasma concentration of H_2_S in healthy animals and humans ranges from 10 to 300 *μ*mol/L [30, 31]. The alteration of H_2_S-producing enzymes and endogenous H_2_S levels are associated with the development of pulmonary fibrosis. In a rat model of bleomycin- (BLM-) induced pulmonary fibrosis, plasma H_2_S content and lung tissue CSE activity (H_2_S production rate) in experimental groups were downregulated by 44% and 27%, respectively, on day 7, while the CSE mRNA level in the lungs treated with BLM was 34% and 143% higher than that in controls on day 7 and day 28, respectively. These results may be due to the compensatory mechanism for the decreased H_2_S in the body [9]. The lung hydroxyproline content, as a marker of collagen deposition, was increased by 43% in BLM-treated group on day 7 and 100% on day 28, along with histologic changes of inflammatory cells infiltration, fibroblast proliferation, and collagen deposition, whereas intraperitoneal injection of sodium hydrosulfide (NaHS, 1.4 and 7 *μ*mol/kg body weight, resp.) twice a day decreased the hydroxyproline content and remarkably attenuated the severity of lung fibrosis [9]. These results were further supported by Cao et al. [10] and Li et al. [32]. Consistent with the BLM-induced pulmonary fibrosis, reduced endogenous H_2_S levels in plasma and lung tissue were observed in another rat model of passive smoking-induced pulmonary fibrosis, accompanied by the upregulation of type I collagen expression and the occurrence of typical histopathological changes of pulmonary fibrosis, whereas NaHS administration at 8 *μ*mol/kg once daily remarkably upregulated H_2_S level and inhibited the passive smoking-induced pulmonary fibrosis [28].

According to the above mentioned studies, exogenous H_2_S (NaHS) is considered to have protective effect against pulmonary fibrosis at a relatively low dose from 1.4 *μ*mol/kg body weight twice daily to 28 *μ*mol/kg once daily [9, 10, 28, 32]. However, high concentrations of H_2_S (50~500 ppm) can cause bronchiolitis obliterans (BO) and pulmonary edema and eventually lead to chronic inflammation and pulmonary fibrosis [2, 6, 33]. Except the protective role of H_2_S proved by extensive studies including animal models and* in vitro* experiments [7–10, 28, 32, 34], the altered expression of endogenous H_2_S-producing enzymes and the levels of endogenous H_2_S in patients with pulmonary fibrosis are unknown yet.

### 2.2. H_2_S and Hepatic Fibrosis

Similar to pulmonary fibrosis, hepatic fibrosis is a dynamic process in response to a variety of stimuli such as ethanol, viral infection, and toxins, leading to the destruction of the architecture of liver parenchyma, followed by excessive ECM deposition, fibrous tissues formation, and cirrhosis (the final pathological stage of hepatic fibrosis) [35, 36]. Three endogenous H_2_S-producing enzymes (CSE, CBS, and MST) are all present in the liver [37]. CSE is expressed in the cytosol of hepatocytes, hepatic stellate cells (HSCs), hepatic artery, portal vein, and the terminal branches of the hepatic afferent vessels, while CBS is mainly expressed in the cytosol of hepatocytes, hepatic artery, and portal vein [2, 38]. MST is predominantly localized in mitochondria and cytosolic fractions of pericentral hepatocytes [39]. CSE and CBS are the primary contributors to H_2_S production in the liver [40]. Under physiological conditions, CSE accounts for 97% of the H_2_S output in liver [40]. The metabolic levels of H_2_S and its producing enzymes were observed to change in human hepatic fibrosis and cirrhosis, as well as in animal and cellular models of hepatic fibrosis [11, 13, 20, 23, 38, 41]. In patients with cirrhosis-induced portal hypertension, plasma H_2_S levels were significantly lower than healthy controls and correlated inversely with the disease severity by Child-Pugh score (42.6 ± 4.7 *μ*mol/L, 33.5 ± 7.7 *μ*mol/L, and 22.2 ± 7.9 *μ*mol/L in group Child-Pugh score of A, B, and C, resp., but 43.5 ± 6.2 *μ*mol/L in control group) [20]. This decrease stemmed from a reduction of CBS and CSE expression/activity [21–23, 42]. Whether schistosomiasis cirrhosis-induced portal hypertension (SPH) model of rabbit or bile duct ligation- (BDL-) or carbon tetrachloride- (CCl_4_-) induced cirrhosis model of rat, both revealed reduced H_2_S level in serum and liver tissues by 20% to 80% [13, 20, 38, 41] and decreased CSE protein expression by approximately 30% to 80% [13, 20, 38, 43]. Meanwhile, plasma H_2_S concentrations showed a clear descending trend during the progression of cirrhosis [13, 20]. These results suggest that endogenous H_2_S generated by H_2_S-producing enzymes might involve the pathogenesis of human and animal hepatic fibrosis.

To further prove the protective role of endogenous H_2_S, the effects of exogenous H_2_S supplementation in the progress of hepatic fibrosis were investigated in animal models [11–13, 38, 44]. NaHS administration significantly elevated the serum levels of H_2_S, decreased the portal pressure, attenuated hyaluronic acid level (HA, a serum fibrosis index), downregulated hepatic hydroxyproline content, reduced the number of collagenous fibers, and eventually alleviated the pathologic features of hepatic fibrosis induced by schistosomiasis, bile duct ligation, or carbon tetrachloride in animal models [11–13, 20, 38, 44]. For example, in cirrhotic rats induced by CCl_4_, intraperitoneal injection with NaHS (10 *μ*mol/kg body weight, every two days for 12 weeks) significantly elevated the serum levels of H_2_S by an average of about 1.33-fold, reduced the mean level of serum HA by 38.6%, decreased both the number of collagenous fibers and the hydroxyproline content in livers by 40%, downregulated the alpha-smooth muscle actin (*α*-SMA, a marker of fibrosis) by 50%, and reduced the portal pressure by about 30% [13]. Similarly, NaHS inhibited CCl_4_-induced liver fibrosis in rats at a dosage of 56 *μ*mol/kg body weight once daily.* In vitro*, HSCs isolated from BDL-induced cirrhosis rats had a 40% downregulated CSE expression and an 80% drop of H_2_S production [38]. Besides, Fan et al. [11, 12] investigated the effects of exogenous H_2_S on HSCs activation by ferric nitrilotriacetate (Fe-NTA, 500 *μ*g/L) and found that incubation with various concentrations of NaHS (0, 100, 200, or 500 *μ*mol/L) resulted in a dose-dependent inhibition in HSC proliferation and induction of G1 phase cell cycle arrest and a downregulated expression of collagen I protein.

These researches above indicate that H_2_S plays a protective role against hepatic fibrosis at a low level of 10~56 *μ*mol/kg/day [13, 38, 44], while high concentration of H_2_S may cause hepatotoxicity [37, 45]. Exposure to a deadly concentration of H_2_S (500~1000 ppm) caused abnormal liver function (elevated ALT and AST) in human, as reported previously [46]. Animal studies also demonstrated different degrees of hepatic damage such as enlarged paled livers and severe hyperemia after H_2_S exposure at high concentrations (63 to 500 ppm) [33].

### 2.3. H_2_S and Renal/Kidney Fibrosis

Renal fibrosis, including glomerular sclerosis and tubulointerstitial fibrosis, is the hallmark of progressive renal disease of virtually any etiology such as glomerular hyperfiltration, hyperperfusion and hyperpressure, and ischemia/reperfusion injury. It is characterized by renal parenchymal cells injury and death, interstitial inflammatory cells infiltration, fibroblasts proliferation and myofibroblast transformation, excessive ECM deposition, and fibrogenesis [47, 48]. Similar to liver and lung, renal tissues express all the three endogenous H_2_S-producing enzymes [39]. CBS is predominantly expressed in renal proximal tubules, while CSE is mainly located in renal glomeruli, proximal tubules, interstitium, and interlobular arteries [14]. Besides, MST is also localized in proximal tubular epithelium in the kidney [39]. CBS and CSE are abundant in renal tissues and produce H_2_S in kidney in a combined manner [49]. Under normal physiological conditions, the expression of CSE protein in kidney is 20-fold higher than that of CBS, appearing to be the main H_2_S-forming enzyme in the kidney [2, 40].

The alterations of endogenous H_2_S metabolism and its producing enzymes in renal fibrosis are well studied in* in vivo* models [14, 15, 19, 50, 51]. Unilateral ureteral obstruction- (UUO-) induced model is a commonly used experimental model for renal interstitial fibrosis, which can be easily manipulated with respect to timing, severity, and duration through surgical intervention [47]. After unilateral ureteral obstruction, CBS and CSE expressions in ureteral obstructive mice kidneys were gradually downregulated in a time-dependent pattern, consistent with decreased plasma and tissue H_2_S levels and aggravated interstitial fibrosis in the kidney after UUO [15, 19]. For instance,the protein expression was downregulated by 30.3%, 62.1%, and 70.5% on days 7, 14, and 21 for CBS, respectively, and decreased by 27.2%, 58.2%, and 74.1% for CSE, accompanied by the time-dependent decrease in plasma H_2_S level by 10.56%, 11.76%, and 22.01% after UUO [19]. Accordingly, the area of tubulointerstitial fibrosis was increased by 13.11-fold on day 7, by 31.35-fold on day 14, and by 55.33-fold on day 21, respectively [19]. Song et al. [14] found that the CBS expression was nearly completely ablated by obstructive injury on day 14, but CSE was increased when compared to the contralateral kidney. Meanwhile, H_2_S production in the obstructed kidney or in plasma was dramatically reduced, along with a significant accumulation of collagen fibrils and an enhanced renal expression of *α*-SMA and fibronectin on day 14 after operation [14]. Notably, the increase in CSE expression in obstructive kidney could be explained by a “compensatory mechanism” as mentioned by the author, attempting to maintain the H_2_S level [14]. These results suggest that the expression of H_2_S-producing enzymes and H_2_S levels in obstructive kidney and plasma reflect the severity of renal interstitial fibrosis in the UUO-induced model. Thus, the endogenous plasma H_2_S might be an ideal biomarker for renal fibrosis.

UUO-induced animal model revealed that exogenous H_2_S could impact the development of renal fibrosis [14, 15, 19, 52]. For instance, Zhao et al. [19] demonstrated that intraperitoneal injection with NaHS (1.4 or 7.0 *μ*mol/kg, twice daily immediately after operation) significantly increased plasma and tissue H_2_S concentration in the obstructive kidney and decreased the area of renal tubulointerstitial fibrosis. Jiang et al. [52] also found that NaHS treatment (89 *μ*mol/kg, i.p. injection once daily, 3 days before surgery and thereafter continuously for 9 days) reduced the development of interstitial fibrosis and the fibrous area in the obstructed kidneys. In addition, NaHS administration also alleviated the pathological changes of renal fibrosis in other models of renal injury triggered by gentamicin-induced nephrotoxicity [53] or streptozotocin-induced diabetic nephropathy [50, 51]. However, the metabolic changes of H_2_S and its producing enzymes as well as its therapeutic potentials in patients with renal fibrosis need to be further studied.

### 2.4. H_2_S and Cardiac Fibrosis

Cardiac fibrosis is a pathological process initiated by some harmful stimuli such as myocardial injury, mechanical stretch, and inflammatory stimuli and followed by the proliferation and migration of cardiac fibroblasts and myofibroblasts transdifferentiation. This process then causes excessive ECM deposition and abnormalities in cardiac structure and function including hypertrophy, failure, and arrhythmias [54, 55]. In mammals, CSE is abundant in heart, vascular smooth muscle, and vascular endothelial cells and is the most relevant H_2_S-producing enzyme in the cardiovascular system [56, 57]. CBS mRNA has been found in endocardium cells and atrial and ventricular myocardial cells [58], yet its protein expression is actually rare in mammalian cardiovascular system [2]. MST is predominantly localized in cardiomyocytes in the heart and in the vascular endothelium, but its contribution to H_2_S production is significantly lower [39, 59].

Growing evidence has suggested that endogenous H_2_S and its producing enzymes involve the development of cardiac fibrosis. Enzymes expressions including CSE and CBS and endogenous H_2_S levels were reduced in different animal models of cardiac fibrosis [17, 25, 60–62]. Simultaneously, decreases in endogenous H_2_S and its producing enzymes were negatively correlated with the severity of cardiac fibrosis [17, 25, 60–62]. For instance, endogenous H_2_S concentration in plasma decreased by about 15% in a rat model of cardiac fibrosis induced by pressure overload, and the hydroxyproline content in the cardiac tissue increased by about 65%, with an extensive deposition of collagen in the left ventricle (LV) tissue [25]. In volume overload-induced mouse model of cardiac fibrosis, endogenous H_2_S production and CSE protein expression in the heart were reduced by 60~80% and 35%, respectively, while the expressions of matrix metalloproteinase- (MMP-) 2/9 and tissue inhibitors of metalloproteinase- (TIMP-) 1/3 were robustly increased, along with a 1.24-fold rise of total hydroxyproline and an increase in collagen deposition [17, 60]. The similar alterations of endogenous H_2_S-producing enzymes and H_2_S were also seen in mouse model of cardiac fibrosis mediated by myocardial infarction induced by ligation of left anterior descending coronary artery. For example, the expressions of CSE and CBS proteins in hearts were decreased by about 80% and 60%, respectively, after myocardial infarction [62], and the plasma H_2_S concentration was significantly decreased to 53.3 ± 2.7 *μ*mol/L compared to the controls (65.1 ± 1.5 *μ*mol/L), while the percentage of fibrosis size to total area of left ventricle in rats of myocardial infarction was more than twofold higher than that in controls (25.7 ± 1.2% versus 12.5 ± 0.5%; *P* < 0.01) [61].

The protective effect of exogenous H_2_S in cardiac fibrosis has been demonstrated in various animal models [17, 18, 25, 60–66]. In a model of spontaneously hypertensive rats (SHR), chronic treatments with NaHS (i.p. 10, 30, and 90 *μ*mol/kg/day, for 3 months) were all effective in reducing indexes of perivascular and interstitial fibrosis in the heart including perivascular collagen area-to-luminal area ratio (PVCA/LA) and collagen volume fraction (CVF) [65]. In pressure overload-induced heart failure model of mouse by transthoracic or abdominal aortic banding, administration of NaHS (po, 30 *μ*mol/L, or i.p. 14 *μ*mol/kg/day) significantly upregulated the levels of endogenous H_2_S in plasma and reduced the deposition of collagen in perivascular and intracardiac parenchymal tissue [25, 64]. Similarly, NaHS (30 *μ*mol/L) or sodium thiosulfate (Na_2_S_2_O_3_, 3 mg/mL) ameliorated the decreased CSE expression, normalized the reduced H_2_S production, mitigated the expressions of MMP-2/9 and TIMP-1/3, and then attenuated the collagen deposition in LV tissues in a volume overload-induced heart failure model of mouse [17, 60]. The role of exogenous H_2_S in alleviating cardiac fibrosis is also confirmed in the mouse model of myocardial infarction induced by ligation of left anterior descending coronary artery [18, 61, 62, 66]. In the NaHS-treated group of rats after myocardial infarction, H_2_S concentration in plasma, CSE protein content, and mRNA expression in LV myocardium were all significantly increased, compared with vehicle-treated group (69.5 ± 4.6 *μ*mol/L versus 53.3 ± 2.7 *μ*mol/L for plasma H_2_S level, 0.66 ± 0.04 versus 0.51 ± 0.03 for CSE protein, and 0.94 ± 0.03 versus 0.72 ± 0.03 for CSE mRNA, resp.) [61]. Simultaneously, NaHS supplementation prevented the increase of MMP-2 and MMP-9 expression in the border zone of infracted tissues on day 14 [18] and decreased ratio of the fibrosis size to total area of LV at the end of the 6th week, compared with vehicle-injected controls (12.5 ± 0.5% versus 25.7 ± 1.2%) [61].

## 3. Protective Mechanisms of H_**2**_S in the Development of Fibrosis 

### 3.1. H_2_S and Oxidative Stress

Oxidative stress, resulting from an increased production of free radicals including reactive oxygen and nitrogen species (ROS and RNS) and an overwhelmed antioxidant defense system, plays a prominent role in the progression of fibrosis [17, 67–70]. ROS mainly consists of superoxide anion, hydroxyl radical (HO^∙^), and hydrogen peroxide, and RNS mainly consists of nitric oxide, nitrogen dioxide, and peroxynitrite. There are two kinds of antioxidant system in our body: one is enzymatic antioxidant system including superoxide dismutase (SOD), catalase (CAT), glutathione peroxidase 1 (GPx1), heme oxygenase 1 (HO-1), and NAD(P)H: quinone oxidoreductase 1 (NQO1). The other is nonenzymatic antioxidant system, including vitamin C, vitamin E, glutathione (GSH), thioredoxin-1 (Trx-1), melatonin, *α*-lipoic acid, carotenoids, and trace elements (copper, zinc, and selenium). Multiple studies revealed that ROS/RNS could activate profibrotic mediators (e.g., protease activated receptor-1/2 (PAR-1/2), a disintegrin and metalloproteinase-12 (ADAM-12), MMP-2/9, and TIMP-1/3) and suppress antifibrotic factors such as TIMP-4 and *β*1-integrin, leading to parenchymal cells apoptosis, fibroblasts activation, and collagen deposition [17, 68, 69, 71]. By directly scavenging oxygen free radicals [15, 72, 73], inhibiting lipid peroxidation [9, 15, 52], modulating the balance of MMPs/TIMPs and ADAM-12/*β*1-integrin axis [17, 59, 63, 64], and activating antioxidant system [15, 52, 63, 65, 73, 74], H_2_S reduces the intracellular redox environment and alleviates oxidative stress-induced damage. Given the important role of oxidative stress in the pathogenesis of fibrosis, it is reasonable to suspect that endogenous H_2_S/H_2_S-producing enzymes pathway inhibits the development of fibrosis by its antioxidative action.

In animal models of lung fibrosis, bleomycin stimulated inflammatory cells to generate excess ROS and then promoted oxidative stress such as lipid peroxidation (LPO), resulting in activation of profibrotic mediators like PAR-2 and MMPs 2/9, apoptosis of epithelial cell, and accumulation of collagen, thus leading to pulmonary fibrosis [71, 75, 76]. NaHS administration attenuated lung malondialdehyde (MDA, a marker of tissue fibrosis) and hydroxyproline formation in a rat model of bleomycin-induced pulmonary fibrosis [9]. In another rat model of pulmonary fibrosis induced by chronic cigarette smoke exposure, H_2_S could significantly decrease cigarette smoking-induced oxidative stress-related indexes like MDA and ROS in serum and lung tissue and enhance the activities of serum SOD and GPx, which is associated with the activation of nuclear factor E2-related factor (Nrf2) and the upregulation of HO-1 and Trx-1 proteins in the lung tissue [28]. In CCl_4_-induced animal model of cirrhosis and Fe-NTA-induced* in vitro* model of hepatic fibrosis, the overproduction of ROS triggers lipid peroxidation, along with deficient antioxidants such as GSH, causes the imbalance between oxidative stress and antioxidant defense, and finally promotes HSCs proliferation and collagen synthesis in liver [11–13, 44, 68, 77]. Exogenous H_2_S inhibits the Fe-NTA-induced elevation of intracellular ROS level and inhibition of proliferation of HSC cells, attenuates the CCl_4_-induced elevation of hepatic MDA level, and decreases in hepatic GSH level, resulting in the reduction of collagen deposition in liver tissue, which are related to the inhibition of phosphorylated p38 MAPK (mitogen-activated protein kinase) and activation of phospho-Akt signaling pathway [11–13]. Similarly, exogenous H_2_S supplement reversed these pathophysiological changes of fibrosis in kidney in animal models through inhibition of oxidative stress and recovery of antioxidant defense system, which may be attributed to the activation of Nrf2 and the upregulation of its downstream targets including HO-1 and NQO1 proteins in the renal tissue [15, 51–53].

In cardiac fibrosis induced by chronic volume/pressure overload or persistent hyperglycemia, H_2_S ameliorates oxidative stress, decreases levels of MMP-2/9, TIMP-1/3, and ADAM-12, and increases the levels of TIMP-4 and *β*1-integrin [17, 59, 60, 63–65]. In addition, H_2_S itself and the increased H_2_S-derived thiols (principally GSH) are involved in the maintenance of enzymatic activation of antioxidant enzymes (e.g., CAT and SOD) in the myocardium [63, 65, 66]. NADPH oxidase 2 and NADPH oxidase 4 (Nox2 and Nox4), as a major source of ROS, play a critical role in profibrotic responses in cardiac fibroblasts and ischemic myocardium; moreover, Nox-generated ROS can mediate the conversion of fibroblasts into myofibroblasts via an extracellular signal-regulated kinase 1/2- (ERK1/2-) dependent signaling pathway; exogenous H_2_S significantly inhibits cardiac Nox2/4 expression, ROS generation, and ERK1/2 phosphorylation [18, 78].

In short, H_2_S can prevent the development of fibrosis by regulating the balance between oxidation and antioxidation and the detailed mechanisms underlying its antioxidant and antifibrotic effects involve the inhibition of phosphorylated p38 MAPK and Nox4-ROS-ERK1/2 signaling pathway and the activation of phospho-Akt and Nrf2-induced antioxidant signaling pathway [9, 11–13, 15, 17, 18, 28, 51–53, 74, 78].

### 3.2. H_2_S and Inflammation

Inflammation has been reported to be the initial stage in the development of fibrosis [79, 80], which causes parenchymal cells apoptosis, fibroblasts proliferation, and ECM deposition, ultimately leading to irreversible fibrotic injury [81, 82]. Treatment with H_2_S significantly decreases the infiltration of inflammatory cells, downregulates proinflammatory cytokines like inducible nitric oxide synthase (iNOS), tumor necrosis factor-*α* (TNF-*α*), interleukin-1 (IL-1), IL-4, IL-6, and IL-8, and then inhibits the progression of fibrosis [10, 13–15, 18, 28, 51, 52, 78].

Inflammatory responses are associated with pulmonary fibrosis [79]. In the process of pulmonary fibrosis induced by bleomycin, different inflammatory cells including neutrophils and eosinophils appeared in the alveoli and interstitium at the early stage, followed by foci of collagen deposition, while large area of fibrosis instead of inflammatory infiltration was observed at the late stage [10]. Similarly, significant fibrosis and inflammatory cell infiltrations also emerged in the lung of smoking rats [28]. Nuclear factor-kappa B (NF-*κ*B) regulates the generation of a lot of proinflammatory cytokines including high-sensitivity C-reactive protein (hs-CRP), TNF-*α*, IL-1, IL-6, and IL-8 and promotes the proliferation of fibroblast and the formation of fibrosis [10, 28, 75, 83]. MAPK, which consists of three major members, ERK1/2, c-Jun N-terminal kinase (JNK), and p38, also plays an important role in regulating inflammation and fibrosis [84]. In addition, Th1/Th2 balance may play a vital role in the processes of inflammation and fibrosis, as Th2 cytokines such as IL-4 mediate inflammatory response and then enhance the fibrotic process by augmenting fibroblasts proliferation and collagen production, while Th1 cytokines like interferon- (IFN-) *γ* have inhibitory effects on fibroblast proliferation and collagen synthesis [85]. Studies have demonstrated that H_2_S can suppress the expression of NF-*κ*B p65 and inflammatory markers like hs-CRP, TNF-*α*, IL-1*β*, and IL-6, inhibit the phosphorylation of MAPKs, and regulate Th1/Th2 balance by elevating the ratio of IFN-*γ*/IL-4, thus alleviating inflammation and delaying the progression of pulmonary fibrosis [10, 28].

Chronic inflammation and the associated regenerative wound healing responses are strongly associated with the development of hepatic fibrosis and cirrhosis [86]. CCl_4_-induced cirrhotic rats showed significantly high levels of serum proinflammatory cytokines including TNF-*α*, IL-1*β* and IL-6, and soluble ICAM-1 (intercellular cell adhesion molecule-1), while simultaneous administration of NaHS resulted in a significant decrease of these cytokines, along with alleviated collagenous fibers in the liver [13]. Interstitial inflammation plays an important role in the priming and progression of renal fibrosis as it can induce apoptosis in tubular cells and promote extracellular matrix production, fibroblast proliferation, and epithelial to mesenchymal transition [80, 81]. Exogenous H_2_S was found to mitigate the renal interstitial inflammatory response through the inhibitions of NF-*κ*B and MAPKs signal pathways and then result in fibrogenesis suppression in kidney [14, 15, 51]. Similarly, supplementation with exogenous H_2_S significantly decreased mRNA and protein levels of inflammatory biomarkers (iNOS, TNF-*α*, ICAM-1, and VCAM-1 (vascular cell adhesion molecule-1)) in the border zone of infracted myocardium tissues and also reduced granulocyte influx into necrotic areas to some extent [18, 78]. Furthermore, studies demonstrated that the heat shock protein “HO-1” had an inhibitory effect on the inflammatory and fibrotic responses in injured myocardium and H_2_S therapy markedly increased HO-1 protein expression in both the ischemic heart and angiotensin II- (Ang II-) stimulated cardiac fibroblasts, accompanied by ameliorative cardiac fibrosis and inflammation in ischemic myocardium [18, 87].

Taken together, H_2_S may exert its anti-inflammatory and antifibrotic effect through inhibiting the activation of NF-*κ*B and MAPKs (p38, JNK, and ERK), as well as upregulating the ratio of Th1/Th2 and the expression of HO-1 protein [10, 14, 15, 17, 28, 51, 87].

### 3.3. H_2_S and Fibroblasts

Numerous studies have shown that fibroblasts proliferation, migration, and differentiation to myofibroblasts, along with excessive ECM production, are key events of fibrosis [18, 47, 88–91]. H_2_S could mediate these inhibitory effects by suppressing the activation of proliferation-related genes, protein kinases, signalling pathways, and ion channels [7, 8, 11, 14, 16, 18, 44].

The migration, proliferation, and myofibroblasts transdifferentiation of lung fibroblasts like MRC5 cells, as well as epithelial-mesenchymal transition (EMT) of alveolar epithelial cells like A549 cells, are closely associated with the pathogenesis of pulmonary fibrosis [7, 8]. Studies have reported that incubation with H_2_S significantly inhibited the proliferation, migration, and myofibroblasts differentiation of MRC5 cells and the EMT of A549 cells through suppressing ERK phosphorylation and (transforming growth factor beta-1) TGF-*β*1-Smad2/3 signaling pathways [7, 8, 92]. In the process of liver fibrosis, HSCs activation is characterized by a transformation from quiescent vitamin A-rich cells to myofibroblasts with enhanced proliferation, fibrogenesis and ECM synthesis, and contractility [89]. NaHS treatment could alleviate hepatic fibrosis by reducing the contractility of HSCs and attenuating ECM deposition through the downregulation of calcium influx and collagen I protein expression in activated HSCs [11]. In addition, TGF-*β*1 is a profibrogenic agent in liver injury and hepatic fibrosis [93, 94]. Shen et al. [44] elucidated that exogenous H_2_S could downregulate TGF-*β*1 expression, prevent HSC activation and proliferation, reduce ECM synthesis, and consequently have antifibrosis effect on liver. For renal fibroblasts, NaHS decreased the cell number and the DNA synthesis of normal rat kidney fibroblasts (NRK-49F), which was associated with the decreased expressions of proliferation-related genes including proliferating cell nuclear antigen (PCNA) and c-Myc [14]. NaHS treatment blocked the transdifferentiation of quiescent renal fibroblasts and tubular epithelial cells into myofibroblasts by inhibiting the TGF-*β*1/Smad3 and MAPKs (ERK, p38, and JNK) signaling pathways in the UUO-induced kidney fibrosis models [14, 15, 95]. Similarly, exogenous H_2_S was found to suppress Ang II-mediated cardiac fibroblast activation and profibrotic activity by repressing Nox4-ROS-ERK1/2 signaling pathway [18]. In addition, NaHS effectively reduced proliferation and myofibroblast transformation of atrial fibroblasts via inhibition of TGF-*β*1 function and the activities of BK_Ca_, I_to_, and IK_ir_ channels [16].

The inhibitory effects of H_2_S on these fibroblasts activation and the underlying mechanisms are summarized as follows: (1) H_2_S downregulates the expressions of proliferation-related genes including PCNA and c-Myc, which are associated with its antiproliferative effect [14]; (2) H_2_S reduces the phosphorylation of MAPKs (p38, JNK, and ERK), increases the phosphorylation of Akt, and thus further suppresses fibroblasts proliferation and migration [7, 8, 12, 51]; (3) H_2_S inhibits myofibroblast transformation of fibroblasts with blockade of TGF-*β*1-Smad signaling pathway [14, 16, 44, 50, 51, 96]; (4) H_2_S suppresses fibroblasts activation by repressing Nox4-ROS-ERK1/2 signaling pathway [18]; (5) H_2_S negatively modulates the activity of calcium and potassium channels through downregulating Ca^2+^ influx, large conductance Ca^2+^-activated K^+^ current (BK_Ca_), transient outward K^+^ current (I_to_), and inwardly rectifying K^+^ current (IK_ir_) in fibroblasts and then reducing fibroblast proliferation and myofibroblast transdifferentiation [11, 16, 97, 98].

### 3.4. H_2_S and Apoptosis

H_2_S has both a proapoptotic effect on fibroblasts and an antiapoptotic effect on parenchymal cells, which depends on the regulation of cell cycle, apoptosis-related factors, and apoptotic signaling pathways, thus interfering with the progress of fibrosis [11, 17, 34, 52, 61, 64, 66]. During the normal wound healing, the number of fibroblasts is reduced through apoptosis [96]. However, it has been reported that fibroblast is resistant to Fas-mediated apoptosis in the process of pulmonary fibrosis [99]. Baskar et al. [34] found that H_2_S propelled MRC5 fibroblast cells towards apoptotic death by inducing DNA damage and cell cycle arrest at G1 phase and activating various apoptosis-related factors, such as p53, p21, ku70, ku8, Bax, and cytochrome c.

In the pathogenesis and development of liver fibrosis, cell cycle arrest and apoptosis of activated HSCs play an important role [11, 12, 100]. Fan et al. [11, 12] found that NaHS induced a significant increase in the percentage of cells in the G1 phase, with a corresponding decrease in the percentage of cells in the S phase, indicating that NaHS inhibited HSC proliferation by inducing G1 phase arrest. Meanwhile, they discovered that Fe-NTA treatment increased the apoptotic rate of HSC-T6 cells, but NaHS administration led to two contradictory results. In the early stage of hepatic fibrosis, oxidative stress contributed to the activation and transformation of quiescent HSCs and simultaneously promoted the proapoptotic activity of HSCs. And during this period, NaHS might inhibit apoptosis through antioxidant effect, with decreased phospho-p38 and increased phospho-Akt proteins [12, 35]. On the contrary, NaHS treatment resulted in a significantly higher apoptotic rate in HSC-T6 cells treated with Fe-NTA in the later stage, which might be due to the increased oxidative stress [11, 12]. The above two phenomena are consistent with a previous study, which has shown that proapoptotic or antiapoptotic activity depends on the stage of fibrosis [100].

H_2_S propels fibroblasts towards apoptosis by inducing DNA damage and cell cycle arrest at G0/G1 phase, as well as stimulating apoptosis-related factors including p53, p21, ku70, ku8, Bax, and cytochrome c [11, 34]. On the other hand, H_2_S reduces parenchymal cells apoptosis by inhibiting TNF-*α*-mediated proapoptotic signaling pathway, regulating the MMP/TIMP axis, and elevating the ratio of antiapoptotic factors [insulin like growth factor-I (IGF-I), Bcl-2] to proapoptotic factors [Fas ligand (Fas-L), Bax, caspases, and cytochrome c], thus eventually leading to resistance to fibrosis [14, 17, 52, 61, 64, 66].

### 3.5. H_2_S and Angiogenesis

As sensitive to anoxia and ischemia, the heart is the most liable to undergo dysfunction or failure after myocardial ischemia or infarction, accompanied by structural abnormalities including cardiomyocyte loss and myocardial fibrotic remodeling in peri-infarct and infarct area [18, 101]. Angiogenesis, referring to the spontaneous blood vessel formation and/or the growth of new blood vessels from preexisting vessels, is a complex biological process characterized by ECM remodeling as well as endothelial cell growth, migration, and assembly into capillary structures [2, 74]. As angiogenesis promotes the delivery of both oxygen and energy substrates for tissue repair after injury, it is vital for various physiological or pathological events, like normal growth and development, wound healing, or repair after myocardial infarction [2, 102]. Interestingly, previous studies have revealed that H_2_S protects against fibrosis by a proangiogenic effect [62, 74, 103]. In different mouse models of cardiac fibrosis induced by myocardial infarction or pressure overload-mediated heart failure, exogenous H_2_S supplement upregulated the expression of vascular endothelial growth factor (VEGF, a proangiogenic factor) and its receptors including tyrosine kinase receptor (flk-1) and fms-like tyrosine kinase (flt-1) as well as the phosphorylation of endothelial NO synthase (eNOS) and the bioavailability of NO but downregulated the expression of antiangiogenic factors such as endostatin, angiostatin, and parstatin, accompanied by augmented vascular density and reduced intermuscular and perivascular fibrosis in heart tissues [62, 74]. The proangiogenic effect of H_2_S is associated with the proliferation and migration of endothelial cell through the activation of several cellular signaling pathways including the PI-3 K/Akt, the MAPKs (e.g., ERK1/2 and p38), ATP-sensitive potassium (K_ATP_) channels, and VEGF-eNOS-NO pathway [74, 103]. Although exogenous H_2_S inhibits the cardiac fibrosis partially by proangiogenic effect, no much evidence is from lung, liver, and kidney fibrosis.

**Figure 1:**
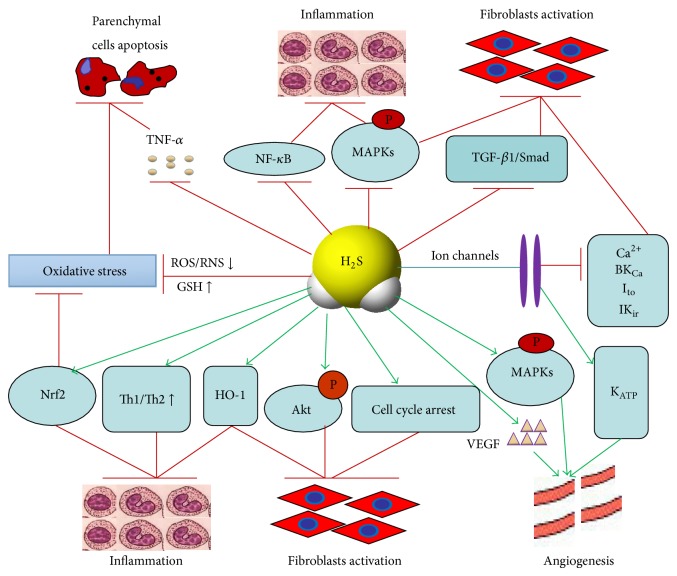
The main mechanisms of H_2_S-mediated protection against fibrosis development. H_2_S plays a complex role in the development of fibrosis. Besides as a reductant to directly scavenge oxygen free radicals, H_2_S exerts its inhibitory effect on fibrosis by anti-inflammation, selectively anti- or proapoptosis, proangiogenic effect, and suppression of fibroblasts activation. Many signaling pathways such as NF-*κ*B, Akt, MAPKs, TGF-*β*1/Smad, and HO-1 and the activity of calcium and potassium channels are involved in the process of antifibrosis of H_2_S. The excitatory effects are denoted by the lines with arrow ends, and the inhibitory effects are indicated by the lines with bar ends. ROS/RNS: reactive oxygen and nitrogen species; GSH: glutathione; TNF-*α*: tumor necrosis factor-*α*; NF-*κ*B: nuclear factor-kappa B; Akt: protein kinase B; MAPKs: mitogen-activated protein kinases; TGF-*β*1: transforming growth factor beta-1; Nrf2: nuclear factor E2-related factor; HO-1: heme oxygenase 1; VEGF: vascular endothelial growth factor; K_ATP_: ATP-sensitive potassium channels.

These protective mechanisms of hydrogen sulfide in the development of fibrosis have been also depicted in Figure 1.

## 4. Concluding Remarks

In this review, we have demonstrated that endogenous or exogenous H_2_S at a physiological or relatively low concentration plays a protective role in the development of fibrosis in lung, liver, kidney, and heart, with its antioxidant, antiapoptotic, anti-inflammatory, proangiogenic properties and its inhibitory effect on fibroblasts activation. These results imply that the endogenous H_2_S-producing enzymes and H_2_S pathway might be a potential therapeutic target for fibrosis. However, we still have a long way to go before a complete understanding of the physical functions of H_2_S in fibrosis pathogenesis. The following issues serve only as examples.

- The degree in endogenous H_2_S-producing enzymes and H_2_S was related to the severity of fibrosis. Although it will be interesting to investigate further whether endogenous H_2_S could be a promising biomarker for fibrosis, it appears to be premature to correlate the measured blood/tissue levels of H_2_S with the severity of fibrosis or the related biological outcomes after H_2_S treatment, as long as the real or exact blood and tissue H_2_S concentrations are still unknown and/or undetectable due to the lack of appropriate techniques.
- As fibrosis can occur in many tissues and organs with a similar pathogenesis, it is reasonable to speculate that endogenous H_2_S-producing enzymes and H_2_S would play an important role in all kinds of fibrosis in the body like pancreatic fibrosis and skin fibrosis.
- Supplementation with exogenous H_2_S such as NaHS not only directly increases H_2_S levels in the body, but also keeps, restores, or even promotes the expression of endogenous H_2_S-producing enzymes like CSE and CBS to produce endogenous H_2_S [15, 18, 19, 50, 51, 60–62, 66]. This positive feedback mechanism is largely unknown and merits further investigation.
- Although H_2_S mitigates the fibrosis development through its antioxidant, antiapoptotic, and anti-inflammatory properties as mentioned above, the exact molecular mechanism is still unknown. Mustafa et al. [104] initially found that H_2_S physiologically modified some proteins' activation by S-sulfhydration. Similar to methylation or acetylation, S-sulfhydration is now recognized as another mode of posttranslational modification. Recent studies found H_2_S can induce Keap1 s-sulfhydration, promote Nrf2 dissociation from Keap1, enhance Nrf2 nuclear translocation, and eventually stimulate mRNA expression of Nrf2-targeted downstream genes such as glutamate-cysteine ligase and GSH reductase to protect against oxidative stress-induced cellular senescence and ischemia/reperfusion injury [105, 106]. Given the important role of Nrf2 in oxidative stress and the development of fibrosis [28], it is reasonable to suspect that H_2_S might modify the Keap1/Nrf2 pathway by S-sulfhydration to protect against fibrosis development.
- Recent research showed organ fibrosis is related to the decrease of autophagy [107, 108]. Impairment of autophagy by TGF-*β*1 or IL-17A promoted fibrogenesis in pulmonary fibrosis [107, 108], while autophagy activation via IL-17A blockage decreased the production of collagen, attenuated fibrosis, and increased survival in the murine model of bleomycin-induced fibrosis [108]. Autophagy plays a complex regulatory pathway in liver fibrosis, with profibrogenic effects relying on the activation of hepatic stellate cells, but with antifibrogenic properties via indirect hepatoprotective and anti-inflammatory properties, as also seen in kidney fibrosis [109–111]. These studies imply that autophagy might involve the pathogenesis of fibrosis. Interestingly, H_2_S can play its biologic roles via autophagy regulation. Exogenous H_2_S can induce/enhance autophagy to inhibit the proliferation of colon epithelial cells or reduce hyperglycemia-induced matrix remodeling by glomerular endothelial cells via signaling pathways of AMP-activated protein kinase (AMPK) and mammalian target of rapamycin (mTOR) [112, 113]. In other conditions, exogenous H_2_S can also suppress excessive activation of autophagy to protect against cigarette smoking-induced left ventricular systolic dysfunction [114] and alleviate traumatic brain injury or ischemia-reperfusion injury [115, 116]. Given the important role of autophagy in the pathogenesis of fibrosis and the regulatory function of H_2_S for autophagy and fibrosis, it is reasonable and interesting to hypothesize that exogenous or endogenous H_2_S might inhibit the development of fibrosis via targeting with autophagy or autophagy-associated signaling pathways.
- The protective role of endogenous or exogenous H_2_S in fibrosis pathogenesis is convincing from various animal models, but much remains unknown of its role in the pathogenesis of human fibrosis. Further clinical studies are needed to translate this potential to clinical use.

## References

[1] Wang R. (2002). Two's company, three's a crowd: can H_2_S be the third endogenous gaseous transmitter?. *The FASEB Journal*.

[2] Wang R. (2012). Physiological implications of hydrogen sulfide: a whiff exploration that blossomed. *Physiological Reviews*.

[3] Wynn T. A. (2008). Cellular and molecular mechanisms of fibrosis. *The Journal of Pathology*.

[4] Wynn T. A. (2007). Common and unique mechanisms regulate fibrosis in various fibroproliferative diseases. *The Journal of Clinical Investigation*.

[5] Parra O., Monsó E., Gallego M., Morera J. (1991). Inhalation of hydrogen sulphide: a case of subacute manifestations and long term sequelae. *British Journal of Industrial Medicine*.

[6] Duong T. X., Suruda A. J., Maier L. A. (2001). Interstitial fibrosis following hydrogen sulfide exposure. *American Journal of Industrial Medicine*.

[7] Fang L.-P., Lin Q., Tang C.-S., Liu X.-M. (2009). Hydrogen sulfide suppresses migration, proliferation and myofibroblast transdifferentiation of human lung fibroblasts. *Pulmonary Pharmacology and Therapeutics*.

[8] Fang L.-P., Lin Q., Tang C.-S., Liu X.-M. (2010). Hydrogen sulfide attenuates epithelial-mesenchymal transition of human alveolar epithelial cells. *Pharmacological Research*.

[9] Fang L., Li H., Tang C., Geng B., Qi Y., Liu X. (2009). Hydrogen sulfide attenuates the pathogenesis of pulmonary fibrosis induced by bleomycin in rats. *Canadian Journal of Physiology and Pharmacology*.

[10] Cao H., Zhou X., Zhang J. (2014). Hydrogen sulfide protects against bleomycin-induced pulmonary fibrosis in rats by inhibiting NF-*κ*B expression and regulating Th1/Th2 balance. *Toxicology Letters*.

[11] Fan H.-N., Wang H.-J., Ren L., Wang C., Li Y.-F., Deng Y. (2013). Protective effects of hydrogen sulfide on oxidativestress and fibrosis in hepatic stellate cells. *Molecular Medicine Reports*.

[12] Fan H.-N., Wang H.-J., Ren L. (2013). Decreased expression of p38 MAPK mediates protective effects of hydrogen sulfide on hepatic fibrosis. *European Review for Medical and Pharmacological Sciences*.

[13] Tan G., Pan S., Li J. (2011). Hydrogen sulfide attenuates carbon tetrachloride-induced hepatotoxicity, liver cirrhosis and portal hypertension in rats. *PLoS ONE*.

[14] Song K., Wang F., Li Q. (2014). Hydrogen sulfide inhibits the renal fibrosis of obstructive nephropathy. *Kidney International*.

[15] Jung K.-J., Jang H.-S., Kim J. I., Han S. J., Park J.-W., Park K. M. (2013). Involvement of hydrogen sulfide and homocysteine transsulfuration pathway in the progression of kidney fibrosis after ureteral obstruction. *Biochimica et Biophysica Acta: Molecular Basis of Disease*.

[16] Sheng J., Shim W., Wei H. (2013). Hydrogen sulphide suppresses human atrial fibroblast proliferation and transformation to myofibroblasts. *Journal of Cellular and Molecular Medicine*.

[17] Mishra P. K., Tyagi N., Sen U., Givvimani S., Tyagi S. C. (2010). H_2_S ameliorates oxidative and proteolytic stresses and protects the heart against adverse remodeling in chronic heart failure. *American Journal of Physiology: Heart and Circulatory Physiology*.

[18] Pan L.-L., Liu X.-H., Shen Y.-Q. (2013). Inhibition of NADPH oxidase 4-related signaling by sodium hydrosulfide attenuates myocardial fibrotic response. *International Journal of Cardiology*.

[19] Zhao D.-A., Liu J., Huang Q., Han Z.-M. (2013). Change in plasma H_2_S level and therapeutic effect of H_2_S supplementation in tubulointerstitial fibrosis among rats with unilateral ureteral obstruction. *Zhongguo Dang Dai Er Ke Za Zhi*.

[20] Wang C., Han J., Xiao L., Jin C.-E., Li D.-J., Yang Z. (2014). Role of hydrogen sulfide in portal hypertension and esophagogastric junction vascular disease. *World Journal of Gastroenterology*.

[21] Ruiz García-Tevijano E., Berasain C., Rodríguez J. A. (2001). Hyperhomocysteinemia in liver cirrhosis: mechanisms and role in vascular and hepatic fibrosis. *Hypertension*.

[22] Look M. P., Riezler R., Reichel C. (2000). Is the increase in serum cystathionine levels in patients with liver cirrhosis a consequence of impaired homocysteine transsulfuration at the level of *γ*-cystathionase?. *Scandinavian Journal of Gastroenterology*.

[23] Fiorucci S., Distrutti E., Cirino G., Wallace J. L. (2006). The emerging roles of hydrogen sulfide in the gastrointestinal tract and liver. *Gastroenterology*.

[24] Ryter S. W., Choi A. M. K. (2011). Gaseous therapeutics in acute lung injury. *Comprehensive Physiology*.

[25] Huang J., Wang D., Zheng J., Huang X., Jin H. (2012). Hydrogen sulfide attenuates cardiac hypertrophy and fibrosis induced by abdominal aortic coarctation in rats. *Molecular Medicine Reports*.

[26] Selman M., King T. E. J., Pardo A. (2001). Idiopathic pulmonary fibrosis: prevailing and evolving hypotheses about its pathogenesis and implications for therapy. *Annals of Internal Medicine*.

[27] Ryu J. H., Colby T. V., Hartman T. E., Vassallo R. (2001). Smoking-related interstitial lung diseases: a concise review. *European Respiratory Journal*.

[28] Zhou X., An G., Chen J. (2014). Inhibitory effects of hydrogen sulphide on pulmonary fibrosis in smoking rats via attenuation of oxidative stress and inflammation. *Journal of Cellular and Molecular Medicine*.

[29] Olson K. R., Whitfield N. L., Bearden S. E. (2010). Hypoxic pulmonary vasodilation: a paradigm shift with a hydrogen sulfide mechanism. *American Journal of Physiology—Regulatory Integrative and Comparative Physiology*.

[30] Whitfield N. L., Kreimier E. L., Verdial F. C., Skovgaard N., Olson K. R. (2008). Reappraisal of H_2_S/sulfide concentration in vertebrate blood and its potential significance in ischemic preconditioning and vascular signaling. *The American Journal of Physiology—Regulatory Integrative and Comparative Physiology*.

[31] Dorman D. C., Moulin F. J.-M., McManus B. E., Mahle K. C., James R. A., Struve M. F. (2002). Cytochrome oxidase inhibition induced by acute hydrogen sulfide inhalation: correlation with tissue sulfide concentrations in the rat brain, liver, lung, and nasal epithelium. *Toxicological Sciences*.

[32] Li H., Liu X.-M., Geng B. (2006). Effect of hydrogen sulfide on Bleomycin-induced pulmonary fibrosis in rats. *Beijing Da Xue Xue Bao*.

[33] Reiffenstein R. J., Hulbert W. C., Roth S. H. (1992). Toxicology of hydrogen sulfide. *Annual Review of Pharmacology and Toxicology*.

[34] Baskar R., Li L., Moore P. K. (2007). Hydrogen sulfide-induces DNA damage and changes in apoptotic gene expression in human lung fibroblast cells. *The FASEB Journal*.

[35] Mormone E., George J., Nieto N. (2011). Molecular pathogenesis of hepatic fibrosis and current therapeutic approaches. *Chemico-Biological Interactions*.

[36] Han Y.-P., Zhou L., Wang J. (2004). Essential role of matrix metalloproteinases in interleukin-1-induced myofibroblastic activation of hepatic stellate cell in collagen. *The Journal of Biological Chemistry*.

[37] Mani S., Cao W., Wu L., Wang R. (2014). Hydrogen sulfide and the liver. *Nitric Oxide*.

[38] Fiorucci S., Antonelli E., Mencarelli A. (2005). The third gas: H_2_S regulates perfusion pressure in both the isolated and perfused normal rat liver and in cirrhosis. *Hepatology*.

[39] Kamoun P. (2004). Endogenous production of hydrogen sulfide in mammals. *Amino Acids*.

[40] Kabil O., Vitvitsky V., Xie P., Banerjee R. (2011). The quantitative significance of the transsulfuration enzymes for H_2_S production in murine tissues. *Antioxidants and Redox Signaling*.

[41] Distrutti E., Mencarelli A., Santucci L. (2008). The methionine connection: homocysteine and hydrogen sulfide exert opposite effects on hepatic microcirculation in rats. *Hepatology*.

[42] Bosy-Westphal A., Petersen S., Hinrichsen H., Czech N., J. Müller M. (2001). Increased plasma homocysteine in liver cirrhosis. *Hepatology Research*.

[43] Taguchi T., Awata S., Nishioka M. (1995). Elevation of cystathionine *γ*-lyase activity in the serum of rats treated with a single dose of carbon tetrachloride. *Industrial Health*.

[44] Shen Q., Qin Z., Lu A. (2012). Preventive effect of exogenous hydrogen sulfide on hepatic fibrosis in rats. *Zhong Nan Da Xue Xue Bao Yi Xue Ban*.

[45] Łowicka E., Bełtowski J. (2007). Hydrogen sulfide (H_2_S)—the third gas of interest for pharmacologists. *Pharmacological Reports*.

[46] Yalamanchili C., Smith M. D. (2008). Acute hydrogen sulfide toxicity due to sewer gas exposure. *The American Journal of Emergency Medicine*.

[47] Chevalier R. L., Forbes M. S., Thornhill B. A. (2009). Ureteral obstruction as a model of renal interstitial fibrosis and obstructive nephropathy. *Kidney International*.

[48] Schnaper H. W., Kopp J. B. (2006). Why kidneys fail: report from an American society of nephrology advances in research conference. *Journal of the American Society of Nephrology*.

[49] Xia M., Chen L., Muh R. W., Li P.-L., Li N. (2009). Production and actions of hydrogen sulfide, a novel gaseous bioactive substance, in the kidneys. *The Journal of Pharmacology and Experimental Therapeutics*.

[50] Yuan P., Xue H., Zhou L. (2011). Rescue of mesangial cells from high glucose-induced over-proliferation and extracellular matrix secretion by hydrogen sulfide. *Nephrology Dialysis Transplantation*.

[51] Zhou X., Feng Y., Zhan Z., Chen J. (2014). Hydrogen sulfide alleviates diabetic nephropathy in a streptozotocin-induced diabetic rat model. *The Journal of Biological Chemistry*.

[52] Jiang D., Zhang Y., Yang M., Wang S., Jiang Z., Li Z. (2014). Exogenous hydrogen sulfide prevents kidney damage following unilateral ureteral obstruction. *Neurourology and Urodynamics*.

[53] Otunctemur A., Ozbek E., Dursun M. (2014). Protective effect of hydrogen sulfide on gentamicin-induced renal injury. *Renal Failure*.

[54] Yue L., Xie J., Nattel S. (2011). Molecular determinants of cardiac fibroblast electrical function and therapeutic implications for atrial fibrillation. *Cardiovascular Research*.

[55] Manabe I., Shindo T., Nagai R. (2002). Gene expression in fibroblasts and fibrosis involvement in cardiac hypertrophy. *Circulation Research*.

[56] Pan L. L., Liu X. H., Gong Q. H., Yang H. B., Zhu Y. Z. (2012). Role of cystathionine *γ*-Lyase/hydrogen sulfide pathway in cardiovascular disease: a novel therapeutic strategy?. *Antioxidants and Redox Signaling*.

[57] Fu M., Zhang W., Yang G., Wang R. (2012). Is cystathionine gamma-lyase protein expressed in the heart?. *Biochemical and Biophysical Research Communications*.

[58] Quéré I., Paul V., Rouillac C. (1999). Spatial and temporal expression of the cystathionine *β*-synthase gene during early human development. *Biochemical and Biophysical Research Communications*.

[59] Sen U., Mishra P. K., Tyagi N., Tyagi S. C. (2010). Homocysteine to hydrogen sulfide or hypertension. *Cell Biochemistry and Biophysics*.

[60] Sen U., Vacek T. P., Hughes W. M. (2008). Cardioprotective role of sodium thiosulfate on chronic heart failure by modulating endogenous H_2_S generation. *Pharmacology*.

[61] Wang X., Wang Q., Guo W., Zhu Y. Z. (2011). Hydrogen sulfide attenuates cardiac dysfunction in a rat model of heart failure: a mechanism through cardiac mitochondrial protection. *Bioscience Reports*.

[62] Qipshidze N., Metreveli N., Mishra P. K., Lominadze D., Tyagi S. C. (2012). Hydrogen sulfide mitigates cardiac remodeling during myocardial infarction via improvement of angiogenesis. *International Journal of Biological Sciences*.

[63] El-Seweidy M. M., Sadik N. A. H., Shaker O. G. (2011). Role of sulfurous mineral water and sodium hydrosulfide as potent inhibitors of fibrosis in the heart of diabetic rats. *Archives of Biochemistry and Biophysics*.

[64] Givvimani S., Munjal C., Gargoum R. (2011). Hydrogen sulfide mitigates transition from compensatory hypertrophy to heart failure. *Journal of Applied Physiology*.

[65] Shi Y.-X., Chen Y., Zhu Y.-Z. (2007). Chronic sodium hydrosulfide treatment decreases medial thickening of intramyocardial coronary arterioles, interstitial fibrosis, and ROS production in spontaneously hypertensive rats. *American Journal of Physiology: Heart and Circulatory Physiology*.

[66] Huang C., Kan J., Liu X. (2013). Cardioprotective effects of a novel hydrogen sulfide agent-controlled release form ulation of S-propargyl-cysteine on heart failure rats and molecular mechanisms. *PLoS ONE*.

[67] Oury T. D., Thakker K., Menache M., Chang L.-Y., Crapo J. D., Day B. J. (2001). Attenuation of bleomycin-induced pulmonary fibrosis by a catalytic antioxidant metalloporphyrin. *American Journal of Respiratory Cell and Molecular Biology*.

[68] Ming-Ju H., Yih-Shou H., Tzy-Yen C., Hui-Ling C. (2011). Hepatitis C virus E2 protein induce reactive oxygen species (ROS)-related fibrogenesis in the HSC-T6 hepatic stellate cell line. *Journal of Cellular Biochemistry*.

[69] Kim J., Seok Y. M., Jung K.-J., Park K. M. (2009). Reactive oxygen species/oxidative stress contributes to progression of kidney fibrosis following transient ischemic injury in mice. *American Journal of Physiology: Renal Physiology*.

[70] Sunami R., Sugiyama H., Wang D.-H. (2004). Acatalasemia sensitizes renal tubular epithelial cells to apoptosis and exacerbates renal fibrosis after unilateral ureteral obstruction. *American Journal of Physiology: Renal Physiology*.

[71] Kalayarasan S., Sriram N., Soumyakrishnan S., Sudhandiran G. (2013). Diallylsulfide attenuates excessive collagen production and apoptosis in a rat model of bleomycin induced pulmonary fibrosis through the involvement of protease activated receptor-2. *Toxicology and Applied Pharmacology*.

[72] Whiteman M., Armstrong J. S., Chu S. H. (2004). The novel neuromodulator hydrogen sulfide: an endogenous peroxynitrite “scavenger”?. *Journal of Neurochemistry*.

[73] Kimura Y., Kimura H. (2004). Hydrogen sulfide protects neurons from oxidative stress. *The FASEB Journal*.

[74] Polhemus D. J., Kondo K., Bhushan S. (2013). Hydrogen sulfide attenuates cardiac dysfunction after heart failure via induction of angiogenesis. *Circulation: Heart Failure*.

[75] Kalayarasan S., Sriram N., Sudhandiran G. (2008). Diallyl sulfide attenuates bleomycin-induced pulmonary fibrosis: critical role of iNOS, NF-kappaB, TNF-alpha and IL-1beta. *Life Sciences*.

[76] Sevanian A., Hochstein P. (1985). Mechanisms and consequences of lipid peroxidation in biological systems. *Annual Review of Nutrition*.

[77] Guo L., Enzan H., Hayashi Y. (2006). Increased iron deposition in rat liver fibrosis induced by a high-dose injection of dimethylnitrosamine. *Experimental and Molecular Pathology*.

[78] Snijder P. M., de Boer R. A., Bos E. M. (2013). Gaseous hydrogen sulfide protects against myocardial ischemia-reperfusion injury in mice partially independent from hypometabolism. *PLoS ONE*.

[79] Pociask D. A., Chen K., Choi S. M., Oury T. D., Steele C., Kolls J. K. (2011). *γδ* T cells attenuate bleomycin-induced fibrosis through the production of CXCL10. *The American Journal of Pathology*.

[80] Eardley K. S., Cockwell P. (2005). Macrophages and progressive tubulointerstitial disease. *Kidney International*.

[81] Lange-Sperandio B., Trautmann A., Eickelberg O. (2007). Leukocytes induce epithelial to mesenchymal transition after unilateral ureteral obstruction in neonatal mice. *The American Journal of Pathology*.

[82] Frangogiannis N. G., Smith C. W., Entman M. L. (2002). The inflammatory response in myocardial infarction. *Cardiovascular Research*.

[83] Cowburn A. S., Deighton J., Walmsley S. R., Chilvers E. R. (2004). The survival effect of TNF-*α* in human neutrophils is mediated via NF-*κ*B-dependent IL-8 release. *European Journal of Immunology*.

[84] Cargnello M., Roux P. P. (2011). Activation and function of the MAPKs and their substrates, the MAPK-activated protein kinases. *Microbiology and Molecular Biology Reviews*.

[85] Kikuchi N., Ishii Y., Morishima Y. (2010). Nrf2 protects against pulmonary fibrosis by regulating the lung oxidant level and Th1/Th2 balance. *Respiratory Research*.

[86] Wang F., Liu S. Y., Du T. P., Chen H., Li Z. Y., Yan J. W. (2014). NF-*κ*B inhibition alleviates carbon tetrachloride-induced liver fibrosis via suppression of activated hepatic stellate cells. *Experimental and Therapeutic Medicine*.

[87] Pachori A. S., Melo L. G., Zhang L., Solomon S. D., Dzau V. J. (2006). Chronic recurrent myocardial ischemic injury is significantly attenuated by pre-emptive adeno-associated virus heme oxygenase-1 gene delivery. *Journal of the American College of Cardiology*.

[88] Wynn T. A. (2011). Integrating mechanisms of pulmonary fibrosis. *The Journal of Experimental Medicine*.

[89] Li D., Friedman S. L. (1999). Liver fibrogenesis and the role of hepatic stellate cells: new insights and prospects for therapy. *Journal of Gastroenterology and Hepatology*.

[90] Porter K. E., Turner N. A. (2009). Cardiac fibroblasts: at the heart of myocardial remodeling. *Pharmacology & Therapeutics*.

[91] Masamune A., Watanabe T., Kikuta K., Shimosegawa T. (2009). Roles of pancreatic stellate cells in pancreatic inflammation and fibrosis. *Clinical Gastroenterology and Hepatology*.

[92] Sime P. J., O'Reilly K. M. A. (2001). Fibrosis of the lung and other tissues: new concepts in pathogenesis and treatment. *Clinical Immunology*.

[93] Liu X., Hu H., Yin J. Q. (2006). Therapeutic strategies against TGF-*β* signaling pathway in hepatic fibrosis. *Liver International*.

[94] Gressner A. M., Weiskirchen R. (2006). Modern pathogenetic concepts of liver fibrosis suggest stellate cells and TGF-*β* as major players and therapeutic targets. *Journal of Cellular and Molecular Medicine*.

[95] Ma F. Y., Sachchithananthan M., Flanc R. S., Nikolic-Paterson D. J. (2009). Mitogen activated protein kinases in renal fibrosis. *Frontiers in Bioscience*.

[96] Chen Y., Wang R. (2012). The message in the air: hydrogen sulfide metabolism in chronic respiratory diseases. *Respiratory Physiology & Neurobiology*.

[97] Soon R. K., Yee H. F. (2008). Stellate cell contraction: role, regulation, and potential therapeutic target. *Clinics in Liver Disease*.

[98] Li G.-R., Sun H.-Y., Chen J.-B., Zhou Y., Tse H.-F., Lau C.-P. (2009). Characterization of multiple ion channels in cultured human cardiac fibroblasts. *PLoS ONE*.

[99] Drakopanagiotakis F., Xifteri A., Polychronopoulos V., Bouros D. (2008). Apoptosis in lung injury and fibrosis. *European Respiratory Journal*.

[100] Chakraborty J. B., Oakley F., Walsh M. J. (2012). Mechanisms and biomarkers of apoptosis in liver disease and fibrosis. *International Journal of Hepatology*.

[101] Van Den Borne S. W. M., Diez J., Blankesteijn W. M., Verjans J., Hofstra L., Narula J. (2010). Myocardial remodeling after infarction: the role of myofibroblasts. *Nature Reviews Cardiology*.

[102] Cochain C., Channon K. M., Silvestre J.-S. (2013). Angiogenesis in the infarcted myocardium. *Antioxidants and Redox Signaling*.

[103] Szabó C., Papapetropoulos A. (2011). Hydrogen sulphide and angiogenesis: mechanisms and applications. *British Journal of Pharmacology*.

[104] Mustafa A. K., Gadalla M. M., Sen N. (2009). H_2_S signals through protein S-Sulfhydration. *Science Signaling*.

[105] Yang G., Zhao K., Ju Y. (2013). Hydrogen sulfide protects against cellular senescence via S-sulfhydration of keap1 and activation of Nrf2. *Antioxidants & Redox Signaling*.

[106] Guo C., Liang F., Shah Masood W., Yan X. (2014). Hydrogen sulfide protected gastric epithelial cell from ischemia/reperfusion injury by Keap1 s-sulfhydration, MAPK dependent anti-apoptosis and NF-*κ*B dependent anti-inflammation pathway. *European Journal of Pharmacology*.

[107] Patel A. S., Lin L., Geyer A. (2012). Autophagy in idiopathic pulmonary fibrosis. *PLoS ONE*.

[108] Mi S., Li Z., Yang H.-Z. (2011). Blocking IL-17A promotes the resolution of pulmonary inflammation and fibrosis via TGF-*β*1-dependent and -independent mechanisms. *The Journal of Immunology*.

[109] Mallat A., Lodder J., Teixeira-Clerc F., Moreau R., Codogno P., Lotersztajn S. (2014). Autophagy: a multifaceted partner in liver fibrosis. *BioMed Research International*.

[110] Ding Y., Choi M. E. (2014). Regulation of autophagy by TGF-*β*: emerging role in kidney fibrosis. *Seminars in Nephrology*.

[111] Principe D. D., Lista P., Malorni W., Giammarioli A. M. (2013). Fibroblast autophagy in fibrotic disorders. *The Journal of Pathology*.

[112] Wu Y. C., Wang X. J., Yu L. (2012). Hydrogen sulfide lowers proliferation and induces protective autophagy in colon epithelial cells. *PLoS ONE*.

[113] Kundu S., Pushpakumar S., Khundmiri S. J., Sen U. (2014). Hydrogen sulfide mitigates hyperglycemic remodeling via liver kinase B1-adenosine monophosphate-activatedprotein kinase signaling. *Biochimica et Biophysica Acta (BBA)—Molecular Cell Research*.

[114] Zhou X., An G., Chen J. (2014). Hydrogen sulfide improves left ventricular function in smoking rats via regulation of apoptosis and autophagy. *Apoptosis*.

[115] Zhang M., Shan H., Chang P. (2014). Hydrogen sulfide offers neuroprotection on traumatic brain injury in parallel with reduced apoptosis and autophagy in mice. *PLoS ONE*.

[116] Wang D., Ma Y., Li Z. (2012). The role of AKT1 and autophagy in the protective effect of hydrogen sulphide against hepaticischemia/reperfusion injury in mice. *Autophagy*.

